# Engaging consumers living in remote areas of Western Australia in the self-management of back pain: a prospective cohort study

**DOI:** 10.1186/1471-2474-13-69

**Published:** 2012-05-11

**Authors:** Helen Slater, Andrew M Briggs, Samantha Bunzli, Stephanie J Davies, Anne J Smith, John L Quintner

**Affiliations:** 1 School of Physiotherapy, Curtin University, Perth, Australia; 2 Curtin Health Innovation Research Institute, Curtin University, Perth, Australia; 3 Department of Health, Government of Western Australia, Perth, Australia; 4 Pain Medicine Unit, Fremantle Hospital and Health Service, Perth, Australia

## Abstract

**Background:**

In Western Australia (WA), health policy recommends encouraging the use of active self-management strategies as part of the co-care of consumers with persistent low back pain (LBP). As many areas in WA are geographically isolated and health services are limited, implementing this policy into practice is critical if health outcomes for consumers living in geographically-isolated areas are to be improved.

**Methods:**

In this prospective cohort study, 51 consumers (mean (SD) age 62.3 (±15.1) years) participated in an evidence-based interdisciplinary pain education program (modified Self Training Educative Pain Sessions: mSTEPS) delivered at three geographically isolated WA sites. Self report measures included LBP beliefs and attitudes (Back Pain Beliefs Questionnaire (BBQ); Fear Avoidance Beliefs Questionnaire (FABQ)), use of active and passive self-management strategies, and health literacy, and global perceived impression of usefulness (GPIU) recorded immediately pre-intervention (n = 51), same day post-intervention (BBQ; GPIU, n = 49) and 3 months post-intervention (n = 25).

**Results:**

At baseline, consumers demonstrated adequate health literacy and elements of positive health behaviours, reflected by the use of more active than passive strategies in self-managing their persistent LBP. Immediately post-intervention, there was strong evidence for improvement in consumers’ general beliefs about LBP as demonstrated by an increase in BBQ scores (baseline [mean (SD): 25.8 (7.6)] to same day post-intervention [28.8 (7.2); P < 0.005], however this improvement was not sustained at 3 months post-intervention. The majority of consumers (86.4%) reported the intervention as very useful [rated on NRS as 7–10].

**Conclusions:**

To sustain improved consumer beliefs regarding LBP and encourage the adoption of more positive health behaviours, additional reinforcement strategies for consumers living in remote areas where service access and skilled workforce are limited are recommended. This study highlights the need for aligning health services and skilled workforce to improve the delivery of co-care for consumers living in geographically isolated areas.

## Background

Persistent low back pain (LBP) is poorly managed and consequently the associated health and economic burden for consumers and for society are substantial [1,2]. For consumers with persistent pain (of which in Australia, LBP represents a significant proportion of the musculoskeletal contribution [1]), less than 10 per cent of patients gain access to effective management, yet according to recent international data, up to 80 per cent could feasibly receive effective care [2,3]. Furthermore, reliable data indicate that the burden of persistent pain for consumers and society can be markedly reduced when available evidence-based management is implemented [2,4].

Many factors contribute to this gap in healthcare for consumers with LBP, including the failure of evidence to be translated into best practice [5-7], an inadequately trained and skilled health workforce [8,9] and a lack of co-ordinated interdisciplinary care [10], the latter being a favoured model for consumers with persistent LBP [11]. To obtain effective care is difficult because persistent pain is often poorly understood by the general community*,* by educators, researchers and health professionals [3,12], often resulting in stigmatization and conditions not being legitimized [13,14]. While there is overwhelming neurobiological evidence of changes in the brain which typically underlie persistent pain [15-18], many health professionals still view consumers with persistent pain within a framework that is biomedical rather than biopsychosocial [19], thereby missing the importance of taking a whole person approach to pain management.

Additional disadvantage exists for consumers with persistent LBP who reside in geographically isolated regions, where there is even less access to evidence-based pain services and community support [12,20]. This is the case for many Australians, where a large landmass is populated with low densities of people, dispersed outside of urban centres, particularly in Western Australia (WA). In this study, we targeted three remote WA locations, which included Kununurra, Albany and Kalgoorlie. Taking the shortest travel route, these towns are situated 3206 kms, 595 kms, and 409 kms respectively away from the state capital Perth. Kalgoorlie and Kununurra lie in the most sparsely populated areas in the state, having a population density of less than 0.1 persons per sq km [21]. Developing innovative models of service delivery based on a contemporary perspective of persistent pain and aligning these models with a skilled workforce, is one strategy targeting the needs of people in these remote areas [22]. Integral to such a model of care for the management of persistent LBP is the facilitation of consumer use of active self-management strategies [23-25], as these are associated with significantly lower rates of health care utilization compared to solely using passive options [23,26].

The focus in this study was the implementation of an interdisciplinary model of care for consumers with LBP living in remote areas of WA using a previously established and consumer-oriented Self Training Educative Pain Sessions (STEPS) program [25]. In a tertiary setting, STEPS has demonstrated significantly reduced wait-times, costs per new patient episode and volume of outpatient appointments and in parallel, an increased utilization of active pain management strategies and increased patient satisfaction [25]. The aim of this project was to deliver and evaluate the effectiveness of a modified STEPS program (mSTEPS) to consumers with persistent LBP living in geographically isolated areas of WA.

## Methods

### Study design/setting/population

Consumers from three remote WA centres (Kununurra, Albany and Kalgoorlie) were invited to attend mSTEPS in their area through flyer advertisements distributed by Rural Health West (rural health medical services) and Arthritis and Osteoporosis WA, the peak advocacy organization for musculoskeletal disease in WA. The three mSTEPS interventions were run during 2010–2011. Those who consented to participate in the evaluation component of the intervention were enrolled in a prospective cohort study. Inclusion criteria required consumers to be able to give voluntary, informed consent for the collection of data and to have persistent LBP. Participants who were not fluent in written and spoken English were excluded from the study. The study was approved by the local Institutional Ethics Review Committees and adhered to the Code of Ethics of the Declaration of Helsinki.

### Participation, consent and anonymity

Eighty consumers and carers registered and attended mSTEPS. Excluding carers from whom data were not collected, 55 consumers were recruited. Of this number, 4 were excluded, 3 of whom were blind and could not complete the data requirements and one of whom did not meet the inclusion criteria (no LBP). The remaining 51 consumers consented to use of their data for the purposes of this study.

At baseline and immediately prior to mSTEPS (i.e.; same day), consenting participants completed a uniquely-coded battery of questionnaires. The educational team remained blinded to the data collection, entry and analysis. At 2.5 months post-intervention, consumers were mailed a post-course questionnaire set with the same unique ID codes, with instructions to complete and to return within 2 weeks (i.e. by 3 months post). Non-responders were contacted by phone on not more than 2 occasions, to request the completion of the post-course questionnaires. In a related study, a subset of 14 of these 51 consumers drawn from each of the 3 locations were also purposively sampled and interviewed in order to better understand their experience of access to health information and services and use of self-management strategies [27]. To contribute to sustainability, the Albany mSTEPs intervention was captured on DVD and provided to mSTEPS consumers post 3 month data collection.

### Study intervention

The mSTEPs program involved the same components as the original STEPS model [25], with the modules delivered in a more compressed format of 6.5 hours over a single day (these components are summarized and presented as Additional file 1).

### Outcome measures

A battery of self-report questionnaires was administered to participants at baseline, and this battery was repeated at 3 months after mSTEPS (Table 1). In addition, on the same day and immediately following mSTEPS, one measure related to back pain beliefs was repeated (Table 1) and participants were also asked to rate the usefulness of the intervention, as described below. To limit responder burden, and given the considerable time required for completion of the baseline battery of questionnaires (30–40 minutes at baseline), the same day post measures were limited to these 2 items. Additionally, it was anticipated that repeating the full battery at this time point would not provide any meaningful data at that time. The battery was designed to align with the core domains and measures of outcomes as outlined in the IMMPACT recommendations [28] and which, while directed at clinical trials, also have broad utility for all clinical pain research [29,30].

**Table 1:** Self report measures administered to consumers with low back pain

**Intervention time points**			
**Self report measures**	**Baseline (pre-intervention)**	**Same day post post -intervention**	**3 months post- intervention**
Demographics	☑		
Pain duration/intensity	☑		☑
Functional limitations	☑		☑
Health care utilisation	☑		☑
Use of self management strategies	☑		☑
DASS^†^	☑		☑
CSQ^§^	☑		☑
BBQ	☑	☑	☑
FABQ^¥^	☑		☑
HeLMS	☑		
GPIU		☑	

A battery of self-report measures was administered to consumers with persistent low back pain who lived in three remote regions of Western Australia. Measures were recorded at baseline (pre-intervention) and measures of specific parameters were repeated at post-intervention (same day and/or at 3 months)

DASS: Depression, Anxiety, Stress Scale (^**†**^2 top loading items from each scale); CSQ: Coping Skills Questionnaire (^**§**^subscale 2: catastrophising); BBQ: back pain beliefs questionnaire; FABQ: fear avoidance beliefs questionnaire (^**¥**^work and physical activity subscales); HeLMS (Health Literacy Measurement Scale); GPIU: Global Perceived Impression of Usefulness.

### Demographic data

Consumers’ age, gender, marital status, country of birth, language spoken, level of education, employment status, medical benefits eligibility, injury compensation status and injury litigation status were recorded.

### Healthcare utilisation

Consumers were asked whether they had ever sought care for their back pain in the past and if so, to indicate from whom, by selecting from pre-specified categories of healthcare practitioners including doctors, allied health professionals and alternative practitioners, using an ‘other’ category [23,25].

### Clinical data

Elements of the Nordic Musculoskeletal Pain Questionnaire [31] were used to confirm LBP prevalence and to measure pain duration. Mean LBP intensities (current; past week; past month) were measured using an 11 point numerical rating scale (NRS) of 0 to 10, with the left anchor 0 = no pain and the right anchor at 10 = maximal imaginable pain [32]. Consumers were asked to self-nominate up to 3 functional activities that they were unable to do or had difficulty with due to their LBP and to rate the level of difficulty using an 11 point numerical rating scale (NRS) of 0 to 10, with the left anchor 0 = unable to perform activity; and the right anchor at 10 = able to perform activity at pre-injury level. As participants were not asked to re-estimate the level of difficulty with the same activity at post mSTEPS, and thus a change in activity-specific disability could not be calculated, we chose to tabulate the frequencies of reported difficulties with commonly listed activities, and to use the data for a baseline sample description only.

### Use of self-management strategies

Consumers indicated their use of active and passive pain coping strategies when responding to the question ‘Which of the following approaches helps to relieve your back pain?’ (Table 2). These coping strategies were based on those originally described by Brown and Nicassio [33] and subsequently represented [23,25] as four categories: active behavioural, active cognitive, passive behavioural and passive conventional. Consumers could select as many strategies as they considered relevant to their persistent LBP. Definitions for these categories have been well documented elsewhere [23,25].

**Table 2:** Use of self management strategies for consumers with low back pain

**Self management strategies**			
Active behavioural	Active cognitive	Passive	Passive conventional
Exercised	Relaxation	Avoided activity	Took medications
Daily walking	Distraction	Rested	Used brace
Corrected posture/stretches	Prayer	Hot bath/shower	Used TENS machine
Worked	Meditation	Hot/cold packs	Physiotherapy treatment (manipulation)
Did usual tasks	Reduced stress	Massage	Chiropractry
Modified activities	Ignored pain	Smoked	Acupuncture
Did small bits often	Improved diet	Drink Alcohol	Procedures (e.g.; needles)
Physiotherapy (Functional rehabilitation)	Mindfulness awareness		Surgery/operations
The middle road (did not under/overdo things	Graded visual imagery		

Consumers with low back pain were asked to indicate their use of self management strategies by choosing from the four categories listed above.

### Pain-related cognitive-behavioural instruments

#### Coping skills questionnaire (subscale 2; catastrophizing)

The catastrophizing subscale of the Coping Skills Questionnaire (CSQ)[34] was used to assess general pain catastrophizing beliefs, as the subscale appears to demonstrate greater utility in terms of examining coping, appraisals, and pain adjustment compared to the composite scores [35]. This subscale lists 6 items which are scored using a 7-point Likert scale with responses 0 indicating ‘never’ to 6 indicating ‘always’. Scores can range from 0–36 with higher scores indicating greater catastrophizing beliefs. The internal consistency of the subscale is reported to be α=0.85 [36].

### Back pain beliefs

Beliefs about the inevitable consequences of future life with low back problems was measured using the Back Beliefs Questionnaire (BBQ)[37,38]. The BBQ consists of 14 items, each of which is rated on a 5 point Likert scale, ranging from 1 (completely disagree) to 5 (completely agree), yielding a possible range of 9 to 45. A higher score indicates more positive beliefs, suggesting better ability to cope with LBP. The internal consistency (α=0.70) and test-retest reliability (ICC = 0.87) of the BBQ have been established previously [38].

### Fear avoidance beliefs

The Fear Avoidance Beliefs Questionnaire (FABQ) [39] was administered to assess fear avoidance beliefs around work and physical activity. Items are rated using a 7 point Likert scale, with 1 indicating ‘completely agree’ to 7 indicating ‘completely disagree’. Two subscales are calculated with a total possible score of between 0–24 for the FABQ-physical and between 0–42 for FABQ-work. For both subscales, a lower score is consistent with fewer fear-avoidance beliefs. Internal consistency for FABQ-physical and FABQ-work were reported as α=0.77 and α=0.88, respectively [39]. Thresholds of > 14 (FABQ-P) and > 29 (FABQ-W) suggest elevated fear avoidance beliefs [40].

### Emotional functioning

A measure of negative emotional functioning was assessed using the Depression, Anxiety and Stress Scale (DASS21) [41]. The rating scale for DASS uses a 4 point Likert scale with 0 (indicating ‘did not apply to me at all’) to 3 (indicating ‘applied to me very much, or most of the time’). As DASS is based on a quantitative dimensional measure of distress along the axes of depression, anxiety (symptoms of psychological arousal) and stress (the more cognitive, subjective symptoms of anxiety), we limited questions to the two top loading items for each subscale, determined through earlier factor analysis [42] and represented these within the rating categories outlined above (i.e.; indicating degree of difference). Questions and loading were as follows: depression: question 10 (.53) and question 21 (.42); for anxiety: question 7 (.46) and question 19 (.43); and for stress, question 1(.38) and question 14(.42) [42].

### Health literacy

Information about the broader elements of health literacy [43] including the ability to seek, understand and utilize health information, was measured using the Health Literacy Measurement Scale (HeLMS) [44,45]. This tool consists of eight independent domains including patient attitudes towards health, understanding health information, social support, socio-economic considerations, accessing general practice healthcare services, communicating with health professionals, being proactive, and using health information. Each of the 29 items is rated on a 5 point Likert scale with the 1 indicating ‘unable to do’ to 5 indicating ‘able to do without difficulty’. By summing and averaging the items for each domain, a score is calculated for each domain.

### Clinical usefulness of the mSTEPs intervention

Consumers used a Global Perceived Impression of Usefulness (GPIU) scale to rate usefulness of the mSTEPs, scored with an 11-point NRS scale, 0 indicating ‘not at all useful’ to 10 indicating ‘extremely useful’ [46].

### Statistical analyses

Demographic variables for participants were described using mean and standard deviation (SD) for continuous variables or frequencies and percentages for categorical variables. The within-subject changes in questionnaire scores from pre to post intervention were analyzed using dependent t-tests. Responses for each HeLMS item were also dichotomized as ‘no difficulty’ (i.e.; a score of 5 on the Likert scale) or ‘any difficulty’ (i.e.; a score of 1–4 on the Likert scale) and the proportion of scores in each category was calculated, consistent with an earlier approach [47]. Statistical significance was set at α = 0.05.

## Results

### Demographic and clinical data

Demographic data are shown in Table 3 and health care utilization regarding LBP is shown in Table 4, while baseline clinical data are presented in Table 5.

**Table 3:** Demographic characteristics of consumers with persistent low back pain

**Characteristic**	
Region in Western Australia	
*Kununurra*	6 (11.8)
*Albany*	25 (49.0)
*Kalgoorlie*	20 (39.2)
Age (years) (N = 50); mean (SD)[min-max]	62.3 (15.1) [27–86]
Gender (Female) (N = 48)	33 (68.8)
English as a first language (N = 51)	47 (92.2)
Born in Australia (N = 50)	31 (62.0)
Highest education level achieved (N = 48)	
*Completed up to 3 years secondary school*	10 (20.8)
*Technical and Further Education / Vocational college*	8 (16.8)
*Completed all 5 years of secondary school*^***§***^	12 (25.0)
*University qualification*	18 (37.5)
Currently employed (N = 49)	21 (42.9)
Medical benefits eligibility^**¥**^ (N = 51)	27 (52.9)
Current insurance claim for injury (N = 49)	3 (6.1)
Seeing/planning to see a solicitor about their LBP (N = 50)	1 (2.0)

Consumers with persistent low back pain were recruited to the modified Self Training Educative Pain Sessions program which was conducted in three remote regions of Western Australia. Demographic data are presented as n (%) unless indicated otherwise

^**§**^ In Western Australia, to complete High School requires 5 years of study; ^**¥**^ The Australian Federal Government funds a scheme which benefits recipients, including low income earners and selected other groups with access to subsidized prescription medicines and a lower Extended Medicare Safety Net threshold, both of which reduce out-of-pocket costs.

**Table 4:** Health care utilisation for consumers with persistent low back pain

**Previous treatment options accessed for LBP (N = 49)**	**N(%)**
General Practitioner (family physician)	45 (91.8)
Physiotherapist	32 (65.3)
Medical Specialist	22 (44.9)
Chiropractor	21 (42.9)
Clinical Psychologist	6 (12.2)
Psychiatrist	5 (10.2)
Acupuncturist	14 (28.6)
Naturopath	3 (6.1)
Osteopath	4 (8.2)
Rehabilitation specialist	5 (10.2)
Other ^**§**^	2 (4.1)

Consumers were asked to indicate what treatment options they had accessed in the past for the management of their persistent low back pain. The list included an option (‘other’ category) to indicate and specify health professionals additional to the below nominated categories

^**§**^Two consumers nominated seeking treatment from a Bowen Therapist.

**Table 5:** Baseline clinical characteristics for consumers with persistent low back pain

**Clinical characteristics**				
Current episode of LBP present more than 3 months (N = 50)	46 (92.0)			
Current episode of LBP Intensity (NRS^†^ 0-10) (mean(sd)):				
*LBP now (N = 51)*	3.5 (2.9)			
*LBP in last week (N = 51)*	5.0 (2.8)			
*LBP in last 4 weeks (N = 51)*	4.8 (2.4)			
Functional limitations due to LBP	**Activity 1** (N = 43)	**Activity 2** (N = 38)		**Activity 3** (N = 30)
*Exercise*	22 (51.2)	13 (34.2)		8 (26.7)
*Bending, lifting, carrying*	8 (18.6)	8 (21.1)		8 (26.7)
*Sustained postures*	7 (16.3)	10 (26.3)		7 (23.3)
*Housework or gardening*	4 (9.3)	7 (18.4)		5 (16.7)
*Hobbies*	2 (4.7)	0		2 (6.7)
Use of self management strategies (count: mean (SD)) *(N = 49)*				
*Active Behavioural (total possible items = 9)*	3.6 (2.2)			
*Active Cognitive (total possible items = 9)*	2.6 (2.1)			
*Passive (total possible items = 7)*	2.4 (1.6)			
*Passive Conventional Medical (total possible items = 8)*	2.0 (1.4)			
Pain-related cognitive-behavioural scales (mean (SD))				
*BBQ; (N = 49); possible score 9-45*	26.5 (7.9)			
*CSQ:(Factor 2 subscale: catastrophising)(N = 49); possible score 0-36*	9.7 (7.9)			
*FABQ-Physical;(N = 50); possible score 0-24*	14.3 (6.4)			
*FABQ-Work; (N = 43);possible score 0-42*	16.0 (11.7)			
Emotional Functioning	0	1	2	3
DASS^**§**^*possible range 0-3*	(did not apply to me at all)	(applied to me some of the time)	(applied to me a good part of the time)	(applied to me most of the time)
*10*^*a*^*: Nothing to look forward to (N = 47)*	33 (70.2)	5 (10.6)	7 (14.9)	2 (4.3)
*22*^*b*^*: Hard to wind down (N = 50)*	13 (26.0)	16 (32.0)	13 (26.0)	8 (16.0)
*25*^*c*^*: Aware of the action of my heart in the absence of physical exertion (N = 45)*	25 (55.5)	9 (20.0)	8 (17.8)	3 (6.7)
*35*^*b*^*: Intolerant of anything that kept me from getting on with what I was doing (N = 48)*	18 (37.5)	16 (33.3)	8 (16.7)	6 (12.5)
*38*^*a*^*Felt that life was meaningless (N = 48)*	35 (72.9)	7 (14.6)	4 (8.3)	2 (4.2)
*41*^*c*^*: Experienced trembling (N = 48)*	29 (60.4)	9 (18.7)	8 (16.7)	2 (4.2)

Measures included pain intensity, pain duration, functional limitations, use of active and passive self management strategies, back pain beliefs (BBQ), catastrophizing beliefs (CSQ), fear avoidance beliefs regarding work (FABQ-W) and physical activity (FABQ-P), and factors which load to depression, anxiety and stress (DASS). Data are presented as n (%) unless indicated otherwise

LBP: low back pain; †VAS: Visual Analogue Scale; ^**§**^ The two top loading items from each of the three self-report scales of the Depression, Anxiety, Stress Scale (DASS) were used and the scales for each questions are identified as follows: ^a^ Depression scale; ^b^ Stress scale; ^c^ Anxiety scale.

### Use of self-management strategies

At baseline, a range of active and passive self-management approaches were reportedly used by consumers to help relieve their LBP (Table 5). On average, more active strategies were employed than passive strategies. The three most frequently nominated strategies within each of the four categories (data not shown) were: (i) active behavioural: did small bits often, modified activity, exercise and corrected posture/stretches (equal); (ii) active cognitive: relaxation, improved diet, ignored pain; (iii) passive: rest, hot bath/shower, massage; (iv) passive conventional: took medication, physiotherapy treatment and surgery/operation.

### Pain-related cognitive behavioural measures

Baseline mean (SD) scores for each belief measure are shown in Table 5.

### Emotional functioning

The majority of responses to the DASS fell into the 0 and 1 categories for all three dimensions (less negative emotional functioning) (Table 5). A small percentage of consumers endorsed either categories 2 and 3 for various items (suggesting more negative emotional functioning).

### Health literacy

While the mean scores for each of the eight domains on the HeLMS ranged from 4.2 to 4.7, suggesting overall adequate health literacy, the range data highlight that some individuals experienced difficulty within some domains (Table 6). To indicate what proportion of consumers had any level of difficulty with an item, versus the proportion who reported no difficulty, a further analysis was undertaken on each domain to examine the proportion of people who scored 1 to 4 on each item, versus those who scored 5.

**Table 6:** The Health Literacy Measurement Scale (HeLMS) was used to measure consumers’ health literacy

**Domain (number/title)**	**Mean (SD)**	**Min-max**	**Range (%) 1-4**^¥^	**Range (%) 5**^§^
1. Patient attitudes towards their health	4.5 (0.6)	3.0 - 5.0	6.0 - 49.0	51.0 - 94.0
2. Understanding health information	4.4 (0.6)	2.3 - 5.0	22.0 - 61.0	39.0 - 78.0
3. Social support	4.5 (0.6)	2.8 - 5.0	22.0 - 51.0	49.0 - 78.0
4. Socioeconomic factors: accessing healthcare services	4.7 (0.5)	2.7 - 5.0	7.8 - 19.6	80.4 - 92.2
5. Accessing general practitioner (GP) healthcare services	4.5 (0.5)	2.7 - 5.0	15.7 - 34.0	66.0 - 84.3
6. Communicating with health professionals	4.2 (0.8)	1.3 - 5.0	24.0 - 70.6	29.4 - 76.0
7. Being proactive	4.4 (0.6)	3.0 - 5.0	24.0 - 58.8	41.2 - 76.0
8. Using health information	4.5 (0.7)	1.75 - 5.0	8.2 - 44.0	56.0 - 91.8

Consumers’ (N = 50) ability to seek, understand and use health information is shown. Mean score (SD) and range data for each of the eight domains are presented. The possible score for each item within a domain ranged from 1: unable to do to 5: able to do without any difficulty

^**¥**^Proportion of consumers who scored 1 to 4 indicating any level of difficulty difficulty with domain items; ^**§**^Proportion of consumers who scored 5, indicating no difficulty with domain items.

### Same day immediately post-intervention evaluation

There was a clinically-important increase in consumer BBQ scores (N = 46) comparing baseline [mean (SD): 25.8 (7.6)] to same day post-intervention [28.8 (7.2)] [mean difference (95% CI): + 3.0 (0.9 to 5.1); P = 0.005] indicating more positive consumer beliefs about back pain. The majority of consumers (86.4%) rated mSTEPs as useful (NRS: 7 to 10), with a small proportion (9.1%) indicating the intervention was moderately useful (NRS: 4 to 6) and only 2 (4.5%) consumers rating the intervention as not that useful (NRS: 0 to 3).

### Baseline to 3 month post-intervention evaluation

Twenty five subjects (49% response rate) responded to the three month postal questionnaire (Table 7). There were no baseline differences in demographic, clinical, health literacy or any belief measure between responders and non-responders.

**Table 7:** Comparison data (mean(SD); baseline and 3 months post-intervention) for responders with persistent low back pain

**Self report measures**	**Baseline**	**3 months post**	**Difference**
	**Mean(SD)**	**Mean(SD)**	**(Mean; 95% CI)**
Self management strategies			
*Active Behavioural (total possible items = 9)*	4.3 (2.4)	4.5 (2.4)	+0.1 (−0.9,+1.1)
*Active Cognitive (total possible items = 9)*	3.0 (2.5)	2.3 (1.9)	−0.7 (−1.5, +0.0)
*Passive (total possible items = 7)*	3.0 (1.7)	2.8 (1.3)	−0.2 (−0.8, +0.4)
*Passive Conventional Medical (total possible items = 8)*	2.3 (1.7)	1.9 (1.7)	−0.4 (−1.4, +0.6)
Pain-related cognitive-behavioural scales			
*BBQ score; ( N = 23)*	27.2 (6.9)	28.4 (6.8)	+1.2 (−1.4, 3.8,)
*CSQ score:(Factor 2 subscale: catastrophising) (N = 20)*	9.7 (7.9)	8.6 (8.1)	−1.2 (−5.0,2.6)
*FABQ-Physical score; ( N = 24)*	14.0 (6.0)	12.3 (6.2)	−1.7 (−3.9, 0.5,)
*FABQ-Work score; (N = 23)*	16.7 (9.7)	17.9 (10.9)	+1.3 (−3.2, 5.7,)

Post mSTEPS, no significant differences were demonstrated in responders’ (N = 23) use of active or passive self management strategies or their pain-related cognitive-behavioural measures

BBQ: Back Beliefs Questionnaire; CSQ: Coping Skills Questionnaire: subscale 2: catastrophizing; FABQ: Fear Avoidance Beliefs Questionnaire.

Analysis of responders alone demonstrated for the same-day evaluation, a similar significant increase in BBQ scores as the whole group [mean difference (95% CI): +2.8 (0.0 to 5.7); P = 0.049], but the improvement was not sustained at 3 months [mean decrease from same-day post (95% CI): - 2.0 (−0.4 to −3.6); P = 0.018]. The number of self-management strategies used by consumers did not change significantly and none of the pain-related cognitive-behavioural measures indicated any significant changes from baseline scores at 3 months post-intervention. Health care utilization and pain intensity measures did not change significantly (data not shown).

## Discussion

This study evaluated the outcomes of delivering a consumer-oriented, evidence-based interdisciplinary pain education intervention, mSTEPS, to consumers with persistent LBP residing in three geographically-remote areas in WA. At baseline, consumers demonstrated scores suggesting positive back pain beliefs, adequate health literacy and positive health behaviours, the latter reflected by the use of more active than passive strategies in self-managing their persistent LBP. Immediately following mSTEPS, consumers’ general beliefs about back pain were significantly more positive indicating a short term improvement in beliefs and the vast majority of consumers rated the program as useful. At 3 months post-mSTEPS intervention, there was no evidence for significant improvement in any baseline measure. This lack of longer term effect may reflect the already high baseline measures or could indicate that additional reinforcement strategies are required in order to sustain improvements in back pain beliefs. Whether a sustained improvement in back beliefs facilitates the adoption of more positive health behaviour is unclear, although it is likely that additional reinforcement strategies are required for this to occur.

In our study, general back pain-related attitudes and beliefs were similar to those reported in other Australian populations with and without LBP [43,48,49] and to other international cohorts [50]. Immediately following mSTEPS, the clinically significant shift towards more positive beliefs about back pain and its consequences was an important outcome, given negative attitudes and beliefs in consumers with persistent LBP can significantly contribute to disability [39,48,51,52]. The magnitude of the positive shift was similar to that previously demonstrated following an Australian population-based intervention designed to improve beliefs about back pain [51,53]. However, the positive shift in the same-day beliefs in the present study was not sustained when examining differences in 3 month-responder scores at baseline (27.2 ± 6.9), same-day (30.3 ± 7.0) and at 3 months post-intervention (28.4 ± 6.8). These consumers demonstrated a significant decrease in scores at 3 months from their significantly improved levels immediately post-course. The pattern of decrease in BBQ scores is unclear, as data were not collected between the same-day post and the 3 month test points. Therefore, we can safely surmise that a brief intervention such as mSTEPS, used as a stand-alone intervention, appears insufficient to generate a sustained improvement in back beliefs and it is likely that additional measures are required to achieve a sustained positive shift in consumer back pain beliefs. Support measures may involve the repetition of simple evidence-based messages using multimedia [53,54]; supplemental educational written materials [50]; reinforcing strategies delivered via internet-based educational approaches [55]; or active management and group-based cognitive-behavioural approaches [56,57]. The use of internet approaches may be particularly well-suited to geographically-isolated consumers with LBP. Furthermore, we suggest that regular community-based group sessions for consumers with LBP with limited access to pain services and community support [12,20], should be considered by health funding bodies, given the available data demonstrating their cost effectiveness and positive health outcomes [57]. Collectively, our findings suggest that it is important to use any transient change in beliefs as a potential ‘therapeutic window’ to encourage the adoption of positive behavioural change, as beliefs and behaviour for consumers with persistent LBP do not necessarily match [43].

In our study cohort, pain catastrophizing scores were lower compared with those previously reported for people with persistent LBP [58], but higher than those reported for a younger WA urban population with persistent LBP and high disability [43]. Half of the consumers in the present study had elevated fear avoidance beliefs scores, similar to those reported in other LBP populations [59,60]. The need for these cognitive and emotional factors to be addressed is highlighted given that catastrophizing and elevated fear avoidance beliefs can interfere with the adoption of beneficial health behaviours [61]. In this context, the use of timely cognitive-behavioural group sessions, which appear to provide reassurance, lessen isolation and enable participants to learn strategies from each other and re-engage earlier into the workforce [57], could align well with the delivery of a community-based LBP management program. The manner in which messages are conveyed to consumers by health professionals should also be considered as our data highlight a proportion of consumers have some level of difficulty understanding health information and communicating with health professionals, while other evidence points to poor quality communication strategies adopted by health professionals in the context of LBP, particularly with consumers who experience persistent LBP [62].

For treatment of their LBP, consumers accessed primarily general practitioners and physiotherapists or chiropractors, possibly reflecting the available services and accessibility. Notably, almost half of the cohort had also consulted a specialist regarding their persistent LBP, and on most occasions, this would have required them to visit a tertiary facility in Perth. The provision of additional services to assist in reinforcing key health messages and to encourage positive health behaviour could be achieved, in part through community-based educational programs combined delivered by ‘virtual’ interdisciplinary teams using novel information technologies, or by the provision of reliable, accessible and evidence-based information resources at the community level, a need previously highlighted by consumers [27]. Such innovative solutions could potentially improve health system efficiencies by providing opportunities to simultaneously upskill both health care professionals and consumers and reduce the need for these consumers to travel to tertiary centres, thereby supporting contemporary health policy directions.

An additional and important component of any optimal model of care for the management of persistent LBP is the facilitation of consumer use of active cognitive and behavioural self-management strategies [23-25]. At baseline, consumers used more active cognitive-behavioural self management strategies than an urban WA population of consumers with persistent spinal pain [25] and more active than passive strategies. Using active strategies to assist in self-managing persistent LBP is important as such approaches substantially reduce the likelihood of consumers having high levels of pain-related disability [23]. Furthermore, adherence to specific self-management strategies can predict reductions in pain, disability and depressive symptoms even after controlling for the moderating effects of catastrophizing, fear-avoidance and pain self-efficacy beliefs [63]. In our study, consumers most frequently employed simple active behavioural approaches that aligned well with their functional limitations, however associated qualitative data from a subset of these consumers suggests that assistance may be required with the adoption and maintenance of long term self management strategies [27]. This may be particularly true for those consumers (up to 71%) reporting difficulty with the broader elements of health literacy, potentially reducing their capacity to engage in positive lifestyle behaviours [27].

Active self management behaviours did not increase significantly at 3 months, although at baseline use of these strategies was already quite high. Several factors which impinge and encourage uptake of self-management strategies have been identified [23,64], and require consideration in the context of our cohort. These can be summarized as follows: 1) limited resources and access to resources; 2) ineffectiveness of pain-relief strategies (evident by those passive conventional strategies chosen by consumers as *not* helping to relieve their LBP and which included surgery, operations, physiotherapy, chiropractic, acupuncture and medication); 3) time constraints and other life priorities ([27,43]; 4) avoiding activity because of fear of pain exacerbation; 5) a lack of tailoring strategies to meet personal needs (possibly incorporating the lack of coordinated interdisciplinary care or limited access to such services); 6) not being able to maintain the use of strategies after the mSTEPS intervention; 7) physical limitations (reflected in consumers’ functional limitations); 8) sub-optimal patient-physician interactions; 9) difficulty in the broader aspects of health literacy.

Standardized evidence-based approaches to chronic health conditions with flexible and tailored self-management and personalized co-care plans could better match consumer needs to resources [65,66], although readiness for change [67] and adherence to these evidence-based approaches is important in optimizing patient outcomes [68,69]. To encourage appropriate service delivery and facilitate the uptake of active self-management and co-care for consumers with persistent spinal pain, we recommend that policy makers and health professionals address the following key considerations: 1) timely provision of integrated evidence-based interdisciplinary co-care [70,71]; 2) ensuring the availability of supportive health professionals [72] and carers; 3) providing a menu of different self-management strategies allowing better matching of consumer profiles with appropriate resources to optimize health outcomes [66,73]; 4) facilitating adherence to self management [63] and providing reinforcement to help encourage the adoption of positive behavioural change [64,74] with reference to an operationalized stages of change framework [75] and readiness to adopt self management [67]. To address these changes requires system plasticity at the individual level (preferably using a w**ho**le **p**erson **e**ngagement model, which we term the HOPE model), in primary care (appropriately skilled interdisciplinary workforce), at a community level (community-based training programs), and at a tertiary level (integrated interdisciplinary co-care). The novel use of information technologies may facilitate such system plasticity for the benefit of health consumers living in geographically-isolated areas.

Our study has several limitations and the applicability of these findings to other populations of consumers with persistent LBP may be limited because of the following factors: a randomized controlled trial would strengthen the study design, as currently the lack of a control group precludes a comparison of the natural time course of outcome measures with change specifically attributed to mSTEPS; data were based on self-report measures; a modest sample size (therefore we cannot control reliably for potential confounding factors like socio-economic status, high and low disability); responder bias (response rate of 49%); and selection bias (consumers in this study were predominantly female (68.8%) and educated (62% had completed high school)). Additionally, consumers were self-referred to mSTEPS and their motivations for attending this educational intervention may differentiate them from consumers not seeking care for persistent LBP.

## Conclusions

The implementation of health policy through a consumer-oriented intervention, set within a contemporary pain management evidence-based framework, can be successfully delivered within primary care. In order to focus management on the whole person and to encourage positive consumer health behaviour, appropriate post-intervention support systems, including access to health services and to appropriately skilled workforce, are recommended. Our findings highlight the need for health delivery change if the current best research in spinal pain is to be effectively and sustainability translated from policy into practice for consumers with persistent LBP living in remote areas.

## Abbreviations

LBP, Low back pain; mSTEPS, Modified Self Training Educative Pain Sessions (STEPS) program; CSQ, Coping Skills Questionnaire; BBQ, Back pain beliefs questionnaire; FABQ, Fear avoidance beliefs questionnaire; DASS, Depression, Anxiety and Stress Scale; HeLMS, Health Literacy Measurement Scale.

## Competing interests

The authors declare they have no competing interests.

## Authors’ contributions

HS, AB, SD, AS, and JQ contributed to the study design and coordination. SB managed the data collection and entry. AS completed the statistical analyses. HS, AB and AS interpreted and reported on the data. All authors contributed to the intellectual content of the manuscript and the manuscript preparation. HS drafted the first and final manuscript versions and acts as guarantor of the paper. All authors contributed to editing the manuscript, contributed to revisions and approved the final manuscript.

## Authors’ information

HS is a clinical researcher and a Specialist Musculoskeletal Physiotherapist with Honorary position at Fremantle Hospital, Pain Medicine Unit. AB is a senior researcher supported by a Fellowship from the Australian National Health and Medical Research Council (NHMRC). SD is a Pain a Medicine Specialist and Head of the Fremantle Hospital, Pain Medicine Unit and Director is Statewide (WA) Pain Medicine Services. AS is a senior researcher and biostatistician who is supported by a Fellowship from Curtin University. JQ is a Rheumatologist and Pain Medicine Specialist. SB is a doctoral student and research assistant.

## Disclosures

The following authors received honoraria for delivering the mSTEPS programs: SD and JQ.

## Pre-publication history

The pre-publication history for this paper can be accessed here:

http://www.biomedcentral.com/1471-2474/13/69/prepub

## Supplementary Material

Additional file 1 Components of the modified Self Training Educative Pain Sessions. Click here for file
