# Supplementary material for: Engaging consumers living in remote areas of Western Australia in the self-management of back pain: a prospective cohort study
Source: BMC Musculoskelet Disord. 2012 May 11;13:69. doi: 10.1186/1471-2474-13-69 (PMC3439262; doi:10.1186/1471-2474-13-69)
Supplement: Additional file 1 — Components of the modified Self Training Educative Pain Sessions. [file 1471-2474-13-69-S1.docx]

# Additional files

### Additional file 1 – Components of the modified Self Training Educative Pain Sessions

### The key modules of the mSTEPS program included: (i) Pacing: introducing pain constructs, forward planning of individually-meaningful activities in a time-contingent manner (pacing, ‘small bits often’ with weekly increments), rather than a pain-contingent manner; educating consumers that ‘chronic pain does not equal ongoing bodily damage’. (ii) Functional motor behaviours: re-educating unhelpful movement patterns and postures within an individually-oriented behavioural framework (O'Sullivan 2005). (iii) Medical options: ‘Sense-making for people in pain: The way in which we make sense of the world so that we can act in it and use of metaphors for understanding pain and communicating complex neurobiology to consumers; discussion of the use of non-addictive prescription pharmacological alternatives. (iv) Pain Approach: use of coping skills, problem-based approaches and the alternative ways that people can respond to pain, acknowledging the potential negative impact of cognitive factors such as catastrophizing on maintaining persistent pain; introduction to using meditation skills and mindfulness [76] to assist in self-regulating, awareness of thoughts, feelings and sensations in the present moment and openness and acceptance towards one’s experiences.
